# Scaphoid Cortical Desmoid in a Snowboarder With Persistent Wrist Pain

**DOI:** 10.7759/cureus.14793

**Published:** 2021-05-01

**Authors:** Sourav Das, George Pujalte, Raphael A. O Bertasi, Dusty Marie Narducci, Peter Murray

**Affiliations:** 1 Family Medicine, Heritage Victor Valley Medical Group, Victorville, USA; 2 Family Medicine, Mayo Clinic, Jacksonville, USA; 3 Anesthesiology and Perioperative Medicine, Mayo Clinic, Jacksonville, USA; 4 Family Medicine, University of South Florida Morsani College of Medicine, Tampa, USA; 5 Orthopedic Surgery, Mayo Clinic, Jacksonville, USA

**Keywords:** scaphoid, desmoid, scapholunate, avulsive cortical irregularity

## Abstract

A 28-year-old man presented to our clinic with persistent left wrist pain. Three months earlier, he had fallen on an outstretched hand while snowboarding. Initial radiographs showed no fractures or dislocations; however, magnetic resonance imaging revealed evidence of a dorsal projection from the scaphoid waist. There was no evidence of a scaphoid wrist fracture, scapholunate ligament disruption, or injury. The final diagnosis was avulsive cortical irregularity or cortical desmoid, which pertains to new bone formation at sites of muscle insertion. The literature has identified cases found in the deltoid, latissimus dorsi, adductor magnus muscles, and gastrocnemius insertion; however, it has never been reported in the scaphoid region. The proposed mechanism, in this case, is the repetitive pull on the radioscaphoid ligament. Avulsive cortical irregularities are benign conditions that mimic malignant conditions radiologically and microscopically. It is therefore important not to mistake this lesion for more worrisome lesions such as osteosarcoma or fibrosarcoma to avoid unnecessary procedures. In the incidental setting, no further imaging is necessary. If the patient presents with pain, atypical radiographical findings, or a suspicion of malignancy, a magnetic resonance image can provide valuable information and confirmation of diagnosis.

## Introduction

Cortical desmoid, also known as avulsive cortical irregularity (ACI) [1], is a benign condition found in new bone formation at the site of muscle insertion [2]. It is usually asymptomatic and incidentally found on radiological images; it should not be misinterpreted as a desmoid tumor, which is an aggressive benign fibroblastic tumor [1]. The knowledge of this entity is of high clinical importance, as it may resemble more aggressive processes such as malignancy, leading to unnecessary procedures [3]. ACI is more commonly found in adolescents [4] at the medial supracondylar femur [5]; however, it has also been reported in the deltoid, latissimus dorsi [6], adductor magnus muscles [4], and ischial tuberosity [7]. Here, we report a unique case of scaphoid ACI found incidentally on magnetic resonance imaging (MRI) after a snowboarder experienced continued wrist pain upon falling on an outstretched hand. An informed consent statement was obtained for this study.

## Case presentation

A healthy, 28-year-old, right-handed man presented to our sports medicine clinic with a history of persistent left wrist pain. He began experiencing pain immediately after falling on an outstretched hand while snowboarding three months earlier, prior to presentation at our clinic. The pain was located mostly over the radial aspect of his left wrist; he rated it as 3 out of 10 in intensity, and associated it with numbness and tingling that radiated down the first web space of his left hand. The pain was initially intermittent but, upon presentation at the clinic, had become persistent. The pain was aggravated by typing, extending the left thumb, and carrying objects up to 30 pounds. The pain was relieved with rest and ibuprofen.

Past medical history included a right scaphoid fracture treated with open reduction and internal fixation with a compression screw. He worked as a consultant, which involved four hours of typing on a computer every day. His hobbies included snowboarding and running.

Three days after the fall, at an outside institution, the physical exam was significant for a bony prominence noted over the volar aspect of the radial side of the left wrist, with no overlying skin changes, swelling, or ecchymosis. Mild pain was elicited by maximal passive ulnar deviation of the left wrist with a point tenderness over the anatomic snuffbox. No other pain was elicited on active, passive, or resisted range of motion testing of his left wrist. Initial posteroanterior and lateral wrist radiographs were performed at the outside institution, three days after the patient’s fall; the patient had only brought the reading, which indicated normal findings (no fractures or dislocations, and joint spaces were maintained). A diagnosis of scaphoid fracture was established, and the patient was advised to rest, apply ice, and elevate the wrist, besides placement of a thumb spica splint for three weeks. Advice on avoidance of exercise, positions that caused pain over the left wrist, and taking ibuprofen as needed for pain, were given.

**Figure 1:**
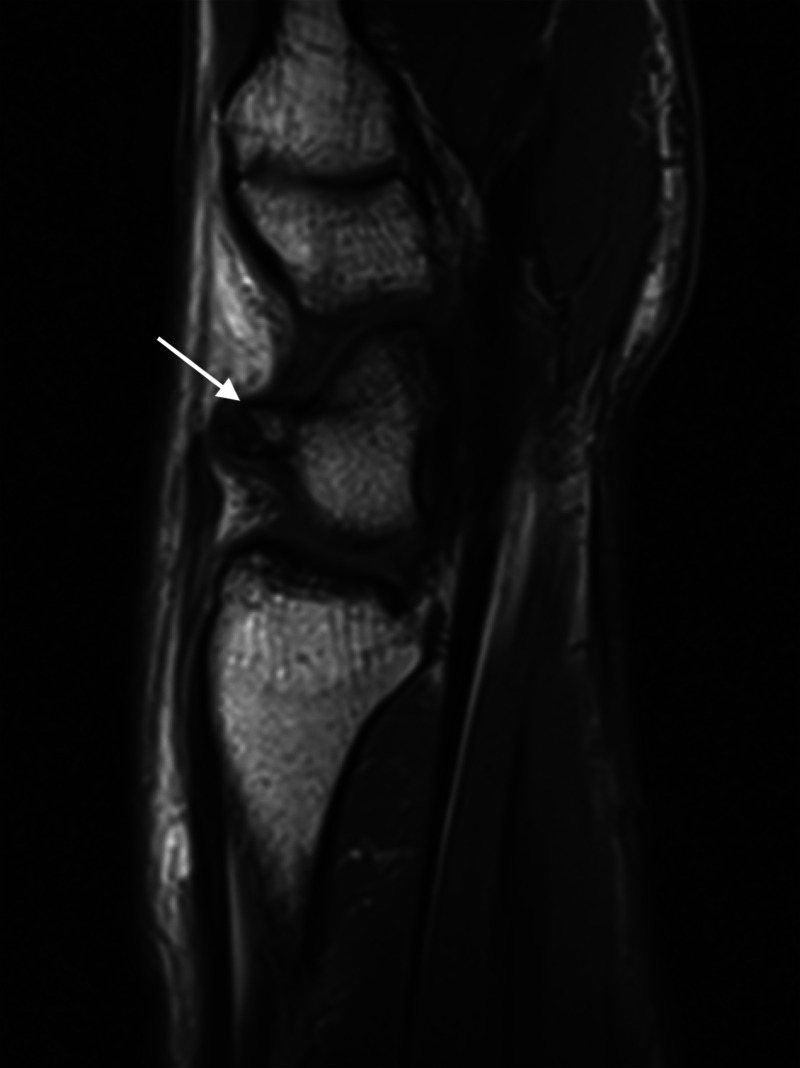
Sagittal Magnetic Resonance Image of the Radial Wrist. An avulsive cortical irregularity of the dorsal scaphoid at the extrinsic ligamentous insertion, likely of the dorsal intercarpal ligament (white arrow). The bones demonstrated no marrow signal abnormality, joint spaces were preserved, and the scapholunate ligament was intact.

After three months, as the pain persisted, the patient presented to our sports clinic. MRI was ordered, given the longstanding nature of continuing pain, physical exam, as well as non-contributory radiographs. MRI was read by an experienced musculoskeletal radiologist, and the finding described as a dorsal projection originating from the scaphoid waist (Figure 1) with no evidence of scaphoid fracture, scapholunate ligament injury, degenerative disease, or dislocation (two experienced hand surgeons concurred with the musculoskeletal radiologist’s interpretation of MRI findings).

Therefore, a diagnosis of ACI at the insertion of the dorsal intercarpal ligaments on the scaphoid was made - a left wrist scaphoid ACI. The patient was advised to rest and avoid any activities that caused pain. The observation was the treatment chosen by the patient, and he was asymptomatic at the three- and nine-month follow-up visits. The patient refrained from snowboarding during that time.

## Discussion

ACI pertains to new bone formation at the site of muscle insertion [2]. The pathogenesis of this lesion is not fully understood. Some researchers believe it is a result of the fibrous cortical defect, while others believe it is caused by the stress of muscular forces at the tendinous attachment site of skeletal muscle [8]. To the best of our knowledge, this is the first report of ACI in the scaphoid confirmed by MRI. As there is no muscle inserting in the scaphoid region, a proposed mechanism, in this case, is repetitive pull on the radioscaphoid ligament. While a single trauma is one that is memorable to the patient, as a snowboarder, he has probably sustained more falls than he can recall with certainty throughout his sporting life, all of which could have contributed to the appearance of the lesion.

ACI is a benign condition that mimics malignancy radiologically and microscopically [9,10]. It is therefore important not to mistake this lesion for osteosarcoma or fibrosarcoma [8] to avoid unnecessary biopsy [3]. As ACI is often found incidentally on x-rays, it can be confusing and subtle when reviewed in conventional radiographs, especially when displacement is minimal [8,11]. Posh and Puckett [12] considered the oblique views of x-rays the best to see a cortex radiolucent defect with periostitis and sclerosis periostitis, which was described as the findings for ACIs.

In the incidental setting, no further imaging is necessary. If the patient presents with pain, atypical radiograph findings, or a suspicion of malignancy, an MRI can provide valuable information and confirmation of diagnosis [8]. The multiplanar capabilities of MRI may be useful in localizing the lesion and providing further details into its nature [11]. In addition, computerized tomography and radionuclide bone scans are also effective imaging methods; however, they expose the patient to ionizing radiation [8,13].

On MRI, ACI is hypointense on T1-weighted images and hyperintense on T2-weighted images, with a dark rim at or near the bony attachment [8]. If edema around the metaphyseal marrow is found in the MRI, suspicion of malignancy or infection should be raised [14]. Bone scintigraphy is another imaging tool used for ACI localization that reveals normal uptake of the radiotracer, except in ACI resulting from stress [12]. When increasing uptake on bone scan, a lesion biopsy is recommended [15].

As ACI is a benign condition, no treatment is needed in asymptomatic patients. There is no consensus regarding the management of patients with pain; however, the treatment usually includes decreased physical activity and pain management [1,16].

In this case, the patient’s lack of pain on passive extension of the wrist perhaps points to an atypical characteristic of this scaphoid injury. The pain on maximal passive ulnar deviation could be a result of increased traction along structures overlying the scaphoid. Interdigital swelling with nerve involvement may have been the cause of the paresthesia noted in the physical exam. As the diagnosis was ACI, no treatment was necessary, and the patient was asymptomatic on the follow-up.

## Conclusions

In conclusion, ACI is usually found incidentally and may mimic malignant conditions radiologically. A plain radiograph with pathognomonic findings of ACI in an asymptomatic patient does not need any additional intervention. However, further evaluation with MRI is required for symptomatic patients, atypical radiograph findings, or high suspicion of malignancy. This case highlighted the importance of knowledge of this benign entity in an unusual location. Primary care and sports medicine physicians should be familiar with this condition to avoid unnecessary procedures and misdiagnosis.
